# Overexpression of Inosine 5′-Monophosphate Dehydrogenase Type II Mediates Chemoresistance to Human Osteosarcoma Cells

**DOI:** 10.1371/journal.pone.0012179

**Published:** 2010-08-16

**Authors:** Jörg Fellenberg, Pierre Kunz, Heiner Sähr, Daniela Depeweg

**Affiliations:** Department of Experimental Orthopedics, Orthopedic University Hospital Heidelberg, Heidelberg, Germany; Mizoram University, India

## Abstract

**Background:**

Chemoresistance is the principal reason for poor survival and disease recurrence in osteosarcoma patients. Inosine 5′-monophosphate dehydrogenase type II (IMPDH2) encodes the rate-limiting enzyme in the *de novo* guanine nucleotide biosynthesis and has been linked to cell growth, differentiation, and malignant transformation. In a previous study we identified *IMPDH2* as an independent prognostic factor and observed frequent *IMPDH2* overexpression in osteosarcoma patients with poor response to chemotherapy. The aim of this study was to provide evidence for direct involvement of IMPDH2 in the development of chemoresistance.

**Methodology/Principal Findings:**

Stable cell lines overexpressing *IMPDH2* and *IMPDH2* knock-down cells were generated using the osteosarcoma cell line Saos-2 as parental cell line. Chemosensitivity, proliferation, and the expression of apoptosis-related proteins were analyzed by flow cytometry, WST-1-assay, and western blot analysis. Overexpression of *IMPDH2* in Saos-2 cells induced strong chemoresistance against cisplatin and methotrexate. The observed chemoresistance was mediated at least in part by increased expression of the anti-apoptotic proteins Bcl-2, Mcl-1, and XIAP, reduced activation of caspase-9, and, consequently, reduced cleavage of the caspase substrate PARP. Pharmacological inhibition of IMPDH induced a moderate reduction of cell viability and a strong decrease of cell proliferation, but no increase in chemosensitivity. However, chemoresistant *IMPDH2*-overexpressing cells could be resensitized by RNA interference-mediated downregulation of *IMPDH2*.

**Conclusions:**

IMPDH2 is directly involved in the development of chemoresistance in osteosarcoma cells, suggesting that targeting of IMPDH2 by RNAi or more effective pharmacological inhibitors in combination with chemotherapy might be a promising means of overcoming chemoresistance in osteosarcomas with high IMPDH2 expression.

## Introduction

Osteosarcoma is the most common primary malignant tumor of bone, typically affecting the long tubular bones of children and adolescents. The prognosis of high-grade osteosarcoma treated with surgery alone has been very poor, with 5-year survival rates below 20% [1]. Major advances in treatment over the past three decades, in particular the introduction of neoadjuvant chemotherapy, have markedly improved the outcome, with long-term relapse-free survival rates ranging from 55% to 75% [2], [3]. However, the remainder of patients respond poorly to chemotherapy with an increased risk of relapse and the development of metastasis. Further efforts to improve patient outcome, for example by means of novel treatment protocols, have not significantly affected overall and disease-free survival of osteosarcoma patients over the past 20 years [4], [5]. The lack of responsiveness to chemotherapy due to intrinsic or acquired chemoresistance is the major reason for poor survival and disease relapse of osteosarcoma patients. However, the mechanisms underlying osteosarcoma chemoresistance remain largely unknown. Therefore, the identification of prognostic factors that allow risk stratification at the time of diagnosis and elucidation of the mechanisms underlying chemoresistance will be pivotal in the development of new therapeutic strategies. In a previous study we identified IMPDH2 (inosine monophosphate dehydrogenase, type II) as independent prognostic factor for the response to chemotherapy in osteosarcoma patients. *IMPDH2* gene expression was significantly elevated in patients with poor response and significantly associated with poor event-free survival [6].

*IMPDH* encodes the rate-limiting enzyme in *de novo* guanine nucleotide biosynthesis, maintaining the cellular guanine deoxynucleotide and ribonucleotide pools needed for DNA and RNA synthesis. IMPDH has been linked to cell growth, differentiation, and malignant transformation [7]–[10]. Two isoforms of IMPDH have been described. Type I is constitutively expressed in normal cells, whereas type II activity has been shown to be increased in proliferating and especially malignant cells [10]–[11]. Thus, IMPDH has been considered an attractive target for immunosuppression as well as antiviral and cancer therapy [12]–[15]. IMPDH inhibitors such as tiazofurin and benzamide riboside have been shown to induce terminal differentiation in a variety of human cancer cells [16], [17] and have been successfully applied in clinical trials [18], [19]. Furthermore, IMPDH2 has been shown to be overexpressed in methotrexate (MTX)-resistant erythroleukemia K562 and human colon cancer cells. Pharmacological inhibition of IMPDH sensitized these cells to MTX treatment, suggesting that IMPDH might be a target for the modulation of chemosensitivity [20], [21].

The aim of the present study was to investigate whether IMPDH2 is directly involved in the development of chemoresistance in osteosarcomas and whether inhibition of IMPDH2 activity or gene expression might usefully improve the outcome of therapy.

Our results demonstrate that *IMPDH2* overexpression induces a strong chemoresistance in osteosarcoma cells which is mediated at least in part by increased expression of anti-apoptotic proteins. Although *IMPDH2* knock-down or pharmacological inhibition of IMPDH2 enzyme activity did not significantly influence the chemosensitivity of wild-type osteosarcoma cells, chemoresistant *IMPDH2*-overexpressing Saos-2 cells were resensitized by IMPDH2 knock-down.

## Results

**Figure 1:**
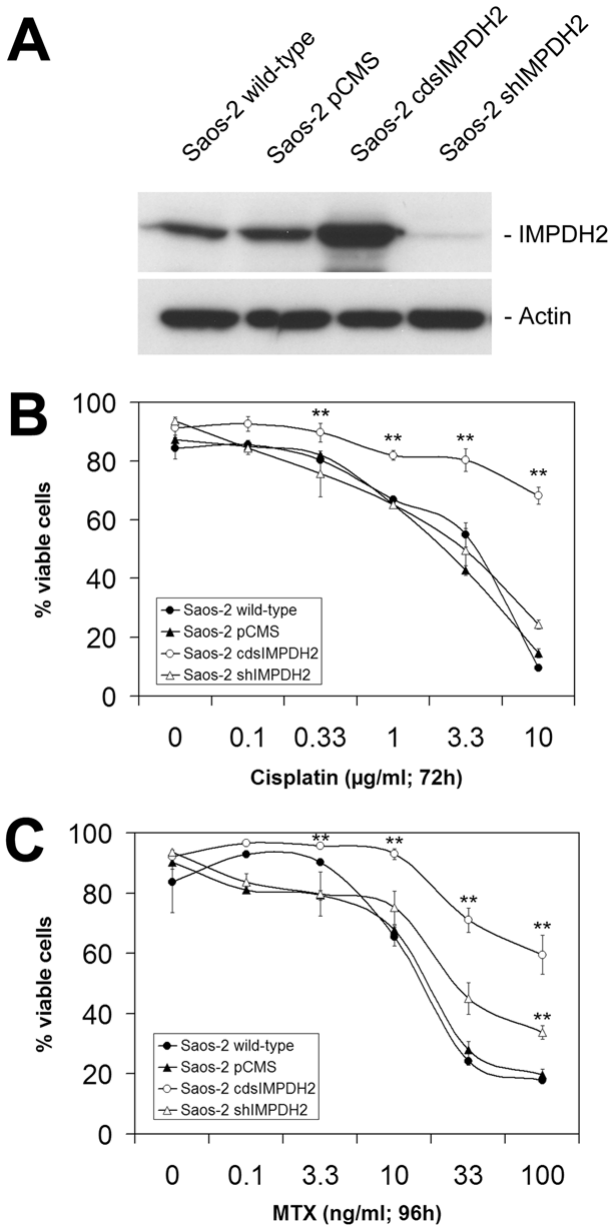
Chemoresistance in *IMPDH2*-overexpressing Saos-2 cells. **A:** Western blot analysis of IMPDH2 expression in Saos-2 wild-type cells, Saos-2 cells transfected with the empty vector (Saos-2 pCMS), Saos-2 cells overexpressing *IMPDH2* (Saos-2 cdsIMPDH2), and Saos-2 cells transfected with a shRNA construct directed against *IMPDH2* (Saos-2 shIMPDH2). **B:** Cell viability of different Saos-2 cell lines after treatment with cisplatin at the indicated concentrations for 72 h. Analyses were performed in triplicate and the results are presented as mean ± SD (** *p*<0.01 compared to Saos-2 wild-type cells). **C:** Cell viability of different Saos-2 cell lines after treatment with methotrexate (MTX) at the indicated concentrations for 96 h. Analyses were performed in triplicate and the results are presented as mean ± SD (** *p*<0.01 compared to Saos-2 wild-type cells).

The observation in our previous study of frequent *IMPDH2* overexpression in osteosarcoma patients with poor response to chemotherapy and the identification of IMPDH2 as an independent prognostic marker for chemotherapy response suggest that IMPDH2 might be directly involved in the development of chemoresistance. To verify this hypothesis we established osteosarcoma cell lines with modulated *IMPDH2* expression either by overexpression of the *IMPDH2* coding sequence in Saos-2 cells (Saos-2 cdsIMPDH2) or by *IMPDH2* knock-down using an shRNA construct specific for *IMPDH2* (Saos-2 shIMPDH2). Western blot analysis of IMPDH2 protein expression in these cell lines showed a marked increase of IMPDH2 expression in Saos-2 cdsIMPDH2 cells and a considerable knock-down of IMPDH2 protein expression in Saos-2 shIMPDH2 cells compared to wild-type cells and cells stably transfected with the empty vector (Fig. 1A).

The analysis of chemosensitivity revealed a strong resistance of *IMPDH2*-overexpressing Saos-2 cdsIMPDH2 cells against cisplatin and methotrexate (Fig. 1B+C). Concerning the calculated IC_50_ values, overexpression of *IMPDH2* induced 118-fold resistance against cisplatin and 14-fold resistance against methotrexate compared to Saos-2 wild-type cells (Table 1). Contrary to our expectations, *IMPDH2* knock-down did not enhance the chemosensitivity of Saos-2 cells (Fig. 1B+C). At high MTX concentrations Saos-2 shIMPDH2 cells even showed a slightly more resistant phenotype rather than the expected sensitive phenotype. We assume that the reduced proliferation rate of IMPDH2 knock-down cells influences the susceptibility of these cells to cytotoxic drugs and that this effect is more pronounced for MTX, which acts much more slowly than cisplatin.

**Table 1:** IC_50_ values of cisplatin and methotrexate in different Saos-2 cell lines.

	Cisplatin	Methotrexate
	(72 h, µg/ml)	(96 h, ng/ml)
Saos-2 wild-type	2.49±0.11	17.72±1.7
Saos-2 pCMS	2.11±0.70	16.97±1.1
Saos-2 cdsIMPDH2	295±26	245±55
Saos-2 shIMPDH2	2.61±0.75	32.13±12

values are presented as mean ± SEM.

**Figure 2:**
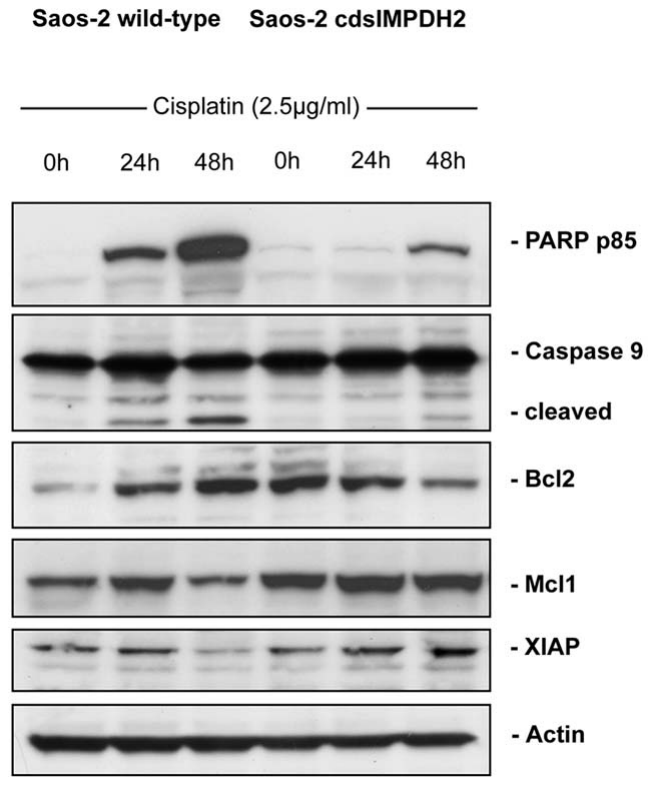
Western blot analysis of different apoptosis-related proteins in Saos-2 wild-type cells and Saos-2 cdsIMPDH2 cells after treatment with cisplatin (2.5 µg/ml) at the indicated time points.

As chemotherapeutic drugs are known to exert their effects mainly through the activation of the mitochondrial apoptosis pathway, we further analyzed the expression of several key players in this pathway in cisplatin-treated Saos-2 wild-type and Saos-2 cdsIMPDH2 cells by western blotting. Cleavage of poly-ADP-ribose polymerase (PARP), a downstream substrate of caspase-9, was markedly reduced in *IMPDH2*-overexpessing cells. Furthermore, the cleavage and therefore the activation of caspase-9 was strongly reduced in these cells (Fig. 2). In addition, untreated Saos-2 cdsIMPDH2 cells showed increased expression of the anti-apoptotic mitochondrial proteins Bcl-2 and Mcl-1 compared to Saos-2 wild-type cells. Upon cisplatin treatment Bcl-2 expression was upregulated in Saos-2 wild-type cells while it was downregulated in *IMPDH2*-overexpressing cells. Mcl-1 expression was slightly upregulated in Saos-2 wild-type cells after 24 h but dropped below control levels after 48 h of treatment. In contrast, Mcl-1 expression in Saos-2 cdsIMPDH2 cells remained unchanged at a high level over the whole observation period (Fig. 2). The expression of the x-linked inhibitor of apoptosis protein (XIAP), a member of the inhibitor of apoptosis family of proteins (IAP), was identical in the untreated cell lines. However, cisplatin decreased the expression of XIAP in wild-type cells while it induced increased expression of XIAP in *IMPDH2*-overexpressing cells (Fig. 2). These findings indicate that the observed chemoresistance of *IMPDH2*-overexpressing osteosarcoma cells may be due at least in part to increased expression of anti-apoptotic proteins, reduced activation of caspase-9, and, as a consequence, decreased cleavage of PARP.

**Figure 3:**
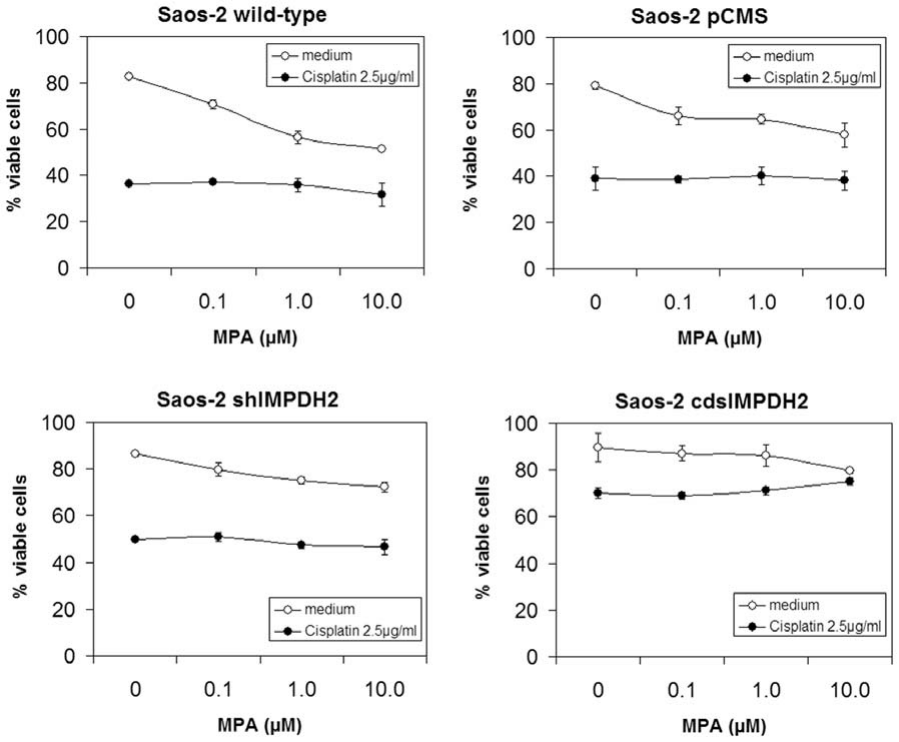
Chemosensitivity of Saos-2 wild-type, Saos-2 pCMS, Saos-2 shIMPDH2, and Saos-2 cdsIMPDH2 cells treated with the IMPDH inhibitor mycophenolic acid (MPA) at the indicated concentrations for 72 h with or without cisplatin (2.5 µg/ml). Analyses were performed in triplicate and the results are presented as mean ± SD.

**Figure 4:**
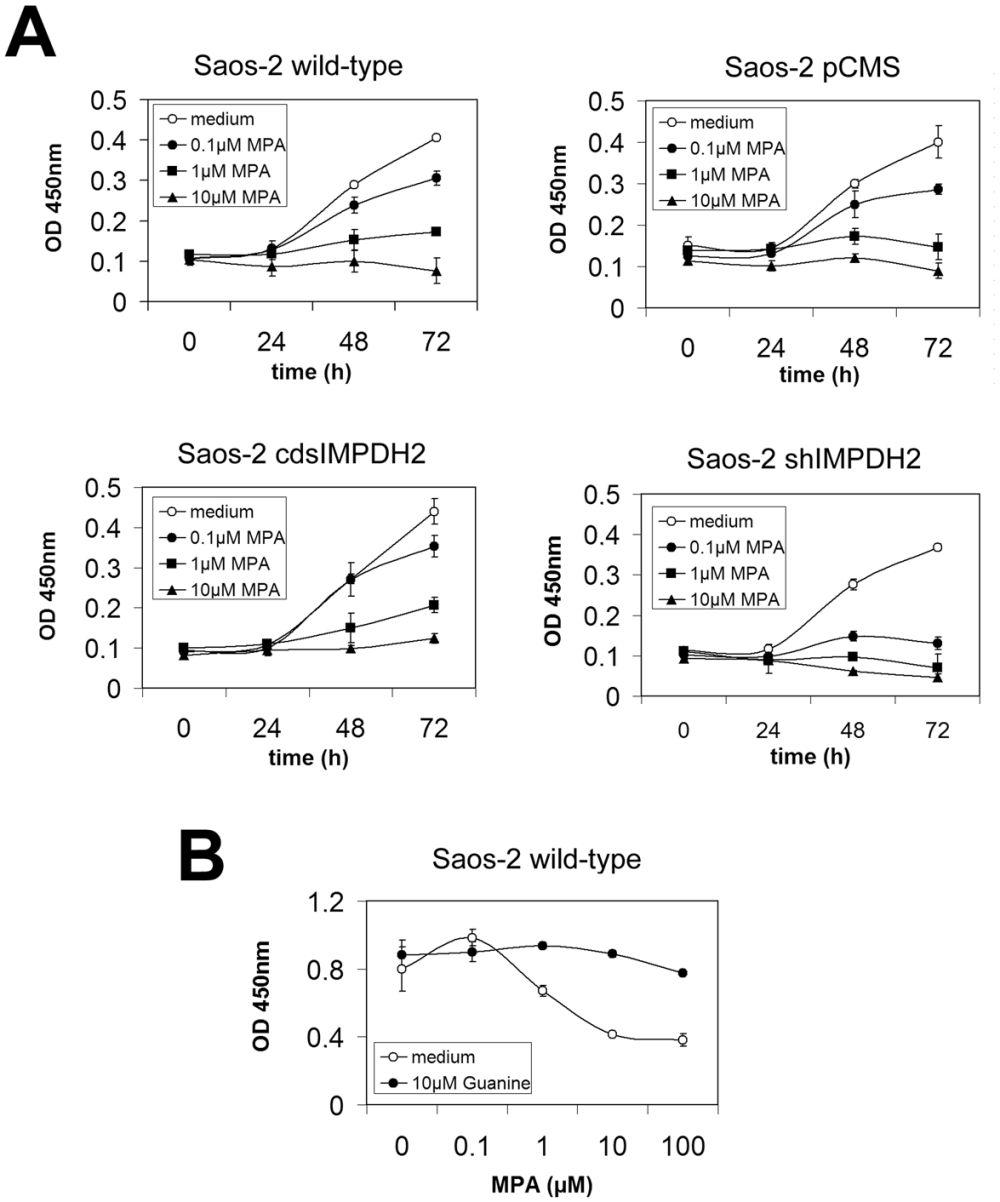
Influence of the IMPDH inhibitor mycophenolic acid (MPA) on cell proliferation of different Saos-2 cell lines. **A:** MPA was added to the culture medium at the indicated concentrations and cell proliferation was determined using WST-1 assay at 0, 24, 48, and 72 h. **B:** Saos-2 wild-type cells were treated with the indicated concentrations of MPA for 48 h with or without the addition of 10 µM guanine. Cell proliferation was analyzed by WST-1 assay at 450 nm. Analyses were performed in triplicate and the results are presented as mean ± SD.

The effects of inhibition of IMPDH2 enzyme activity on cell viability and chemosensitivity were evaluated using mycophenolic acid (MPA), an IMPDH inhibitor with a five times higher affinity to IMPDH2 than to the isoform IMPDH1. MPA alone induced concentration-dependent cell death in Saos-2 wild-type, Saos-2 pCMS, and Saos-2 shIMPDH2 cells. In Saos-2 cdsIMPDH2 cells only the highest MPA concentration induced a slight decrease in cell viability. However, MPA did not influence the chemosensitivity of any of the tested cell lines to cisplatin (Fig. 3). As the susceptibility of cells to treatment with chemotherapeutic drugs is highly dependent on cell growth, we analyzed the effects of MPA on cellular proliferation. MPA inhibited cell proliferation in a concentration-dependent manner in all tested Saos-2 cell lines (Fig. 4A).

To verify that the observed antiproliferative effect of MPA was due to a depletion of guanine nucleotides caused by the inhibition of IMPDH, Saos-2 wild-type cells were treated with MPA with or without the addition of guanine. Guanine almost completely abolished the antiproliferative effects of MPA, indicating a specific inhibition of IMPDH enzyme activity (Fig. 4B).

**Figure 5:**
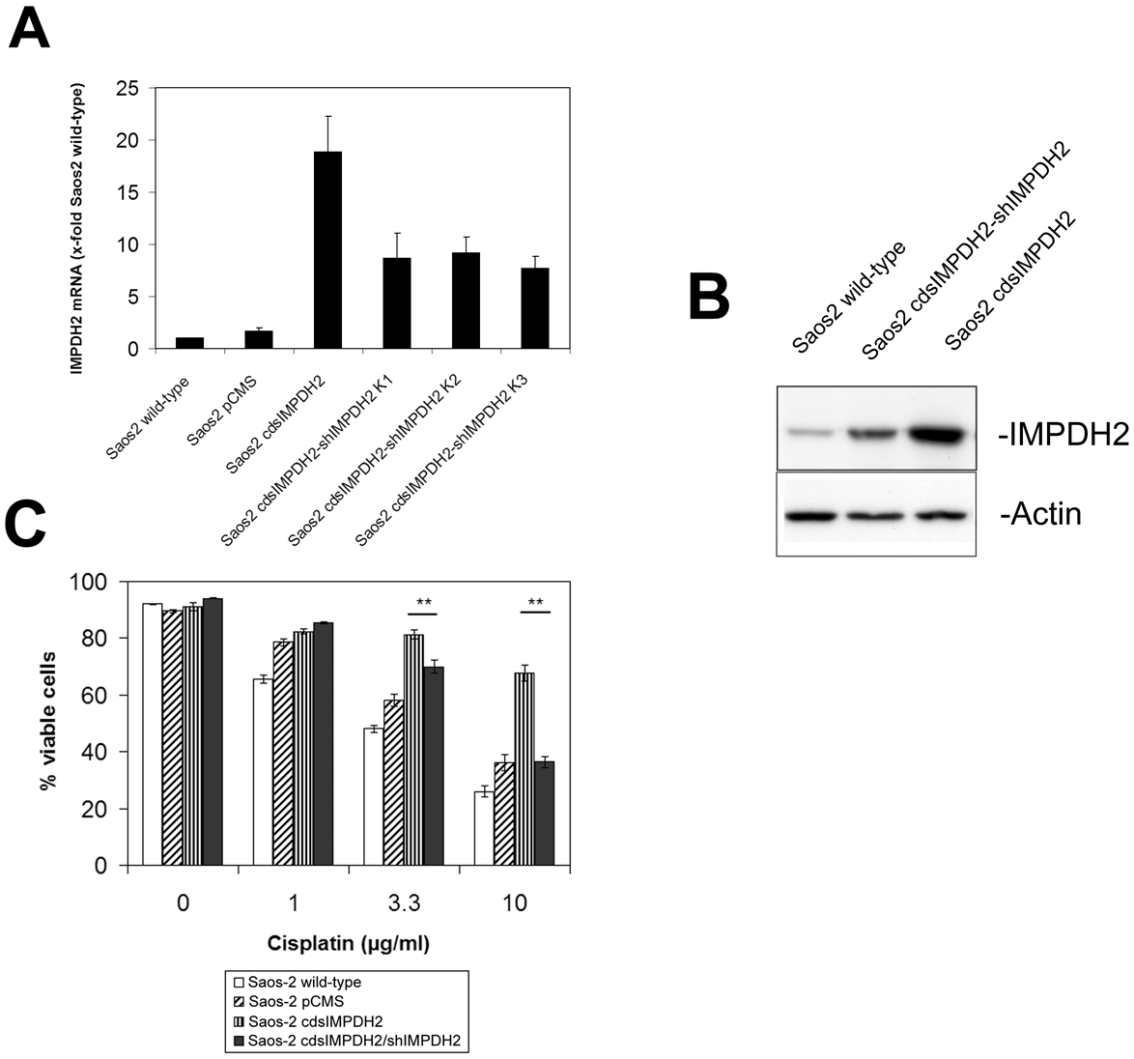
Chemosensitivity of Saos-2 cdsIMPDH2 cells cotransfected with an *IMPDH2-*specific shRNA construct. **A:** Stable Saos-2 cdsIMPDH2 cells were cotransfected with three different shRNA constructs directed against *IMPDH2*. Stable cell lines were generated, and *IMPDH2* mRNA expression was analyzed by quantitative PCR and compared to that in Saos-2 wild-type, Saos-2 pCMS, and Saos-2 cdsIMPDH2 cells. **B:** Western blot analysis of IMPDH2 expression in Saos2 cdsIMPDH2 cells cotransfected with an IMPDH2 specific shRNA construct. **C:** Analysis of chemosensitivity of Saos-2 wild-type, Saos-2 pCMS, Saos-2 cdsIMPDH2, and Saos-2 cdsIMPDH2 cells stable cotransfected with shRNA construct specific for *IMPDH2*. Cells were treated with cisplatin at the indicated concentrations for 72 h before cell viability was analyzed by propidiumiodide staining. Analyses were performed in triplicate and the results are presented as mean ± SD. (** *p*<0.01).

Since the pharmacological inhibition of IMPDH2 did not influence the chemoresistance of *IMPDH2*-overexpressing Saos-2 cells, we analyzed whether reduction of *IMPDH2* gene expression is sufficient to provoke resensitization of these cells. For this purpose we cotransfected Saos-2 cdsIMPDH2 cells with three different shRNA constructs specific for *IMPDH2* and generated stable cell lines. *IMPDH2* mRNA levels were 52–60% lower in these cell lines than in the parent Saos-2 cdsIMPDH2 cells (Fig. 5A). Upon treatment with cisplatin, cotransfection of shIMPDH2 significantly decreased the amount of viable cells compared to Saos-2 cdsIMPDH2 cells. At high cisplatin concentrations the chemosensitivity of these cells was comparable to that of Saos-2 pCMS control cells (Fig. 5B).

## Discussion

The existence or development of intrinsic or acquired chemoresistance represents the principal reason for poor survival and disease recurrence in osteosarcoma patients. Unfortunately, the mechanisms underlying osteosarcoma chemoresistance remain largely unknown. However, knowledge of the mediators that contribute to chemoresistance is pivotal to the identification of high-risk patients and the development of new therapeutic strategies. In order to identify prognostic factors for chemotherapy response we previously screened osteosarcoma cell lines for drug-regulated genes. Among other genes, we observed upregulation of *IMPDH2* in response to cytotoxic drugs [22]. We further screened osteosarcoma biopsies for *IMPDH2* gene expression and correlated these data with the patients' response to chemotherapy as well as their overall and event-free survival. Expression of *IMPDH2* was frequently increased in the subgroup of patients with poor response to chemotherapy and turned out to be an independent prognostic factor significantly associated with chemotherapy response and event-free survival [6]. These data suggested that IMPDH2 might be directly involved in the development of chemoresistance. In fact, the findings of the present study demonstrate that overexpression of *IMPDH2* in osteosarcoma cells induces strong chemoresistance to cisplatin and methotrexate, two drugs frequently used for osteosarcoma therapy. As chemotherapeutic drugs exert their cytotoxic effects mainly through the activation of the mitochondrial apoptosis pathway, we investigated the expression of several apoptosis-related factors. Untreated *IMPDH2*-overexpressing Saos-2 cells displayed increased expression of the anti-apoptotic proteins Bcl-2 and Mcl-1 as well as upregulation of XIAP upon cisplatin treatment. As a consequence, activation of caspase-9 and cleavage of the caspase substrate PARP were markedly reduced in these cells upon treatment with cisplatin, demonstrating IMPDH2-mediated inhibition of the mitochondrial apoptosis pathway. Interestingly, the IMPDH inhibitor mycophenolate mofetil (MMF) has been shown to induce caspase-dependent apoptosis in myeloma cells. Moreover, the pro-apoptotic mitochondrial proteins Bak and Bax have been shown to play an important role in the regulation of apoptotic cell death which is strongly associated with the depletion of the intracellular GTP pool [23]. Thus, the IMPDH-dependent regulation of intracellular GTP-pools seems to be crucial for the balance of anti- and pro-apoptotic proteins, which for their part influence the susceptibility of the cell to apoptotic stimuli, including cytotoxic drugs.

As catalyst of the rate-limiting step in the *de novo* synthesis of guanine nucleotides, IMPDH2 has been identified as an important regulator of cell proliferation [9]. In particular, proliferating lymphocytes are strongly dependent on the *de novo* synthesis of nucleotides, making IMPDH an attractive target for immunosuppressive therapies. IMPDH inhibitors such as MMF and its active compound MPA are thus widely used as immunosuppressive agents. Likewise, rapidly proliferating neoplastic cells are characterized by a high demand on IMPDH-mediated nucleotide synthesis. Expression of *IMPDH*, particularly the type II isoform, has been shown to be significantly increased in many types of malignancies, making IMPDH2 also an attractive target for cancer therapy [10]–[12], [24], [25]. Several studies have already demonstrated the cytotoxic effects of IMPDH inhibitors and their high potential as anticancer drugs [26]–[28]. Phase II/III trials of IMPDH inhibitors such as tiazofurin and benzamide riboside have been conducted with very promising results, although studies were terminated due to neurotoxic side effects [18], [19], [29]. Although the essential role of IMPDH in cancer cell proliferation has been extensively studied, little is known about the involvement of this enzyme in the regulation of chemosensitivity. Increased *IMPDH* mRNA levels have been detected in MTX-treated and MTX-resistant human colon cancer and erythroleukemia cells. Inhibition of IMPDH significantly increased the sensitivity of the resistant cell lines to MTX, indicating that targeting IMPDH might constitute a promising means of minimizing the development of resistance [20], [21]. We did not detect an increase in chemosensitivity in osteosarcoma cells treated with the IMPDH inhibitor MPA. Likewise, knock-down of *IMPDH2* gene expression did not sensitize Saos-2 wild-type cells to cisplatin or methotrexate. Because of these unexpected observations and the fact that cytotoxic drugs preferentially act on rapidly proliferating cells, we assumed that the antiproliferative actions of IMPDH2 inhibitors counteract their effects on chemosensitivity. In fact, MPA induced a strong dose-dependent decrease in cell proliferation with an expected higher sensitivity of *IMPDH2* knock-down cells and a lower sensitivity of *IMPDH2*-overexpressing cells. Besides cell proliferation, the *p53* status of the analyzed Saos-2 cells might contribute to the failure of MPA to induce chemosensitivity. Saos-2 cells have a *p53* null genotype and were chosen because *p53* mutations and *p53* inactivation are common features of osteosarcomas. The *p53* gene functions as a key regulator of the apoptotic program. It is activated in response to cellular stress and exerts its well-documented pro-apoptotic functions mainly in a transcription-dependent manner. The IMPDH inhibitor MPA has been shown to activate and stabilize p53, which in turn mediates cell cycle arrest and apoptosis in response to guanine nucleotide depletion [30]–[32]. These data suggest that a functional p53 pathway is required for the induction of apoptosis in response to nucleotide depletion caused by IMPDH inhibitors. While pharmacological inhibition of IMPDH2 activity did not significantly alter the chemosensitivity of the analyzed cells, *IMPDH2*-overexpressing cells could be resensitized by cotransfection of an *IMPDH2*-specific shRNA construct. In association with the observed decrease in *IMPDH2* gene expression, sensitivity to cisplatin increased, reaching the sensitivity levels of control cells at high cisplatin concentrations.

Altogether, we were able to demonstrate the induction of strong chemoresistance in osteosarcoma cells by overexpression of *IMPDH2*. The observed chemoresistance is mediated at least in part by upregulation of anti-apoptotic proteins, leading to inhibition of the mitochondrial apoptotic signaling pathway. Pharmacological inhibition of IMPDH2 did not enhance chemosensitivity, probably due to the strong antiproliferative effects of the inhibitor or the absence of functional p53. However, reduction of *IMPDH2* gene expression in *IMPDH2*-overexpressing cells resensitized these cells to cytotoxic drugs. As *IMPDH2* overexpression is frequently observed in osteosarcoma patients with poor response to chemotherapy, targeting of IMPDH2 by RNAi or more effective inhibitors in combination with chemotherapy might provide an effective synergistic treatment to overcome chemoresistance in osteosarcomas. It is clear that this hypothesis is still at a very early stage of development but add an interesting point of discussion and explanation of the observed effects.

## Materials and Methods

### Cell culture and drug treatment

The human osteogenic sarcoma cell line Saos-2 was originally obtained from the American Type Culture Collection (Rockville, USA). Cells were maintained in RPMI 1640 (Lonza GmbH, Wuppertal, Germany) containing 2 mM L-glutamine and 25 mM HEPES and supplemented with fetal calf serum (Biochrom, Berlin, Germany), and 100 U/ml penicillin/streptomycin (Lonza GmbH) at 37°C in a humidified 5% CO_2_ atmosphere. Cells were kept in a logarithmic growth phase and split following treatment with trypsin/EDTA (Lonza GmbH), washed in PBS, and replated in fresh culture medium. For drug treatment, cisplatin (Sigma-Aldrich, Taufkirchen, Germany) was dissolved in N,N-dimethylformamide at a concentration of 15 mg/ml and added to the culture medium in the indicated concentrations. Methotrexate was dissolved in 0.01N NaOH and further diluted in PBS to a concentration of 10 mg/ml. Mycophenolic acid (Sigma-Aldrich) was dissolved in methanol at a concentration of 50 mM and added to the culture medium in the indicated concentrations.

### Transfection and generation of stable cell lines

For overexpression of *IMPDH2* the full coding sequence of *IMPDH2* was cloned into the mammalian expression vector pCMS-EGFP (Clontech, Germany) and verified by sequencing. For gene knock-down a commercially available shRNA expression vector expressing the following IMPDH2-specific 29-mer shRNA was used: 5′-ATAGCCTCCATTCGTATGAGAAGCGGCTT-3′ (Origene, Rockville, USA). In both cases Saos-2 cells were transfected by electroporation using 10^5^ cells and 0.5 µg plasmid and the microporator MP-100 (PeqLab, Erlangen, Germany). Forty-eight hours after transfection, medium was replaced by fresh medium containing 600 µg/ml geniticin (Sigma-Aldrich) for cells transfected with the pCMS-EGFP vector or 1 µg/ml puromycin (Sigma-Aldrich) for cells transfected with shRNA constructs. Stable clones were selected for at least 4 weeks before single colonies were picked and analyzed for *IMPDH2* expression by quantitative PCR and western blot.

### Western blot analysis

Cells were washed in PBS, lyzed in RIPA buffer (Santa Cruz, Heidelberg, Germany) containing protease inhibitor cocktail (Santa Cruz), incubated for 1 h at 4°C, and centrifuged at 12000 *g* for 10 min. to remove cellular debris. Total protein concentrations were determined by BCA-assay (Pierce, Rockford, USA), and 10 µg of total protein was subjected to gel electrophoresis. Proteins were separated on a 10% polyacrylamide gel and transferred to Immobilon-P membranes (Millipore, Schwalbach, Germany). After blocking in PBS supplemented with 5% skim milk (Sigma-Aldrich) and 0.05% Tween 20 (Sigma-Aldrich) membranes were incubated overnight at 4°C with one of the following primary antibodies at the indicated dilutions: IMPDH2 (1∶500) (Atlas Antibodies, Stockholm, Sweden), PARP p85 (1∶1000) (Promega, Mannheim, Germany), caspase-9 (1∶1000) (Cell Signaling Technology, Danvers, USA), bcl-2 (1∶250) (Calbiochem, San Diego, USA), mcl-1 (1∶250) (Santa Cruz), XIAP (1∶1000) (Cell Signaling Technology), and actin (1∶5000) (BD Transduction Laboratories, Heidelberg, Germany). After incubation with the primary antibody, membranes were washed three times with PBS containing 0.1% Tween 20 and incubated for 1 h at room temperature with 5000-fold diluted peroxidase conjugated goat anti-rabbit IgG (Santa Cruz) or goat anti-mouse IgG. Proteins recognized by the antibody were visualized with LumiLight western blotting substrate (Roche Diagnostics, Mannheim, Germany) according to the manufacturer's instructions.

### Quantitative PCR

Two micrograms of total RNA extracted with RNeasy Mini kit (Qiagen, Hilden, Germany) were reverse transcribed using Sensiscript (Qiagen) and 10 µM oligo-dT primer for 2 h at 37°C in a total volume of 20 µl. Quantitative real-time PCR was performed in a LightCycler instrument (Roche Diagnostics) in a total volume of 20 µl using the Absolute SYBR Capillary mix (Thermo Scientific, Dreieich, Germany) and 1 µl of cDNA as template. Samples were heated to 95°C for 15 min followed by 40 cycles of denaturation at 95°C for 5 s, annealing at 58°C for 15 s, and extension at 72°C for 20 s. After the last cycle, a melting curve analysis was performed to verify the specificity of the amplified PCR products. The amount of PCR product was calculated using an external standard curve and LightCycler software. Calculated gene expressions were normalized on the basis of the ß-actin (ACTB) expression in the corresponding samples.

### Analysis of chemosensitivity and cell proliferation

Cell viability was quantified by propidium iodide staining and subsequent flow-cytometric analysis. For drug treatment 7.5×10^4^ cells were seeded in a 24-well plate 24 h before treatment. After incubation for the indicated time, adherent and detached cells were collected, washed in PBS, centrifuged, and resuspended in PBS supplemented with 1% FCS and 2.5 µg/ml propidium iodide (Invitrogen, Karlsruhe, Germany). Samples were directly analyzed on a FACScalibur cytofluorimeter using the CellQuest software (Becton Dickinson, Hamburg, Germany).

For the analysis of cell proliferation 2.5×10^3^ cells were seeded in a 96-well plate. After 0, 24, 48, and 72 h, medium was replaced with fresh medium containing 1/10 volume of cell proliferation reagent WST-1 (Roche Diagnostics) and incubated for 2 h at 37°C before the absorbance at 450 nm was quantified in a spectrophotometer.

### Statistics

Statistical analysis was done using the two-tailed Student's *t*-test with *p* = 0.01 considered the upper limit of statistical significance. Data are presented as mean ± SD.
